# Brown Algae Potential as a Functional Food against Hypercholesterolemia: Review

**DOI:** 10.3390/foods10020234

**Published:** 2021-01-24

**Authors:** Rebeca André, Rita Pacheco, Mafalda Bourbon, Maria Luísa Serralheiro

**Affiliations:** 1 BioISI–Instituto de Biossistemas e Ciências Integrativas, Faculdade de Ciências, Universidade de Lisboa, 1749-016 Lisboa, Portugal; raandre@fc.ul.pt (R.A.); ripacheco@fc.ul.pt (R.P.); mafalda.bourbon@insa.min-saude.pt (M.B.); 2 Área Departamental de Engenharia Química, Instituto Superior de Engenharia de Lisboa, Av. Conselheiro Emídio Navarro, 1959-007 Lisboa, Portugal; 3 Unidade I&D, Grupo de Investigação Cardiovascular, Departamento de Promoção da Saúde e Doenças Não Transmissíveis, Instituto Nacional de Saúde Doutor Ricardo Jorge, 1649-016 Lisboa, Portugal; 4 Departamento de Química e Bioquímica, Faculdade de Ciências, Universidade de Lisboa, Campo Grande, 1749-016 Lisboa, Portugal

**Keywords:** brown algae, polysaccharides, phlorotannins, peptides, cholesterol, LDL-c, HDL-c, HMG-CoA, NPC1L1

## Abstract

Brown algae have been part of the human diet for hundreds of years, however, in recent years, commercial and scientific interest in brown algae has increased due to the growing demand for healthier diet by the world population. Brown algae and its metabolites, such as carotenoids, polysaccharides, phlorotannins, and proteins, have been associated with multiple beneficial health effects for different diseases, such as cardiovascular diseases, one of the main causes of death in Europe. Since high blood cholesterol levels are one of the major cardiovascular risks, this review intends to provide an overview of current knowledge about the anti-hypercholesterolemic effect of different brown algae species and/or their isolated compounds.

## 1. Introduction

Seaweeds are macroalgae used in different sectors, such as agricultural, horticultural, cosmetics, and food industries. It has been recognized that the novel and potentially bioactive components that algae present make them a good source of healthy food [1,2]. World seaweed production doubled between 2005 and 2015. Globally, in 2016, seaweed products were valued at USD 10.6 million, and it is estimated that in 2025 the value of global seaweed products will reach USD 26 million [3]. Asia and the Pacific region dominate 60% of the world algae market, followed by Europe and the Americas [3]. Seaweeds have been used as part of the human diet for thousands of years. Archaeological evidence shows that in Chile it has been used for the last 14,000 years [1], and in Japan and China, there are written records describing the use of seaweed that date back over 2000 years [4,5]. Nowadays, in Europe, seaweed consumption is increasing, not only because people are becoming interested in the uses of natural products, but also because it is seen as a healthy and nutritious “superfood”, which is sold preserved dry, fresh, frozen, canned, or salted [3,6]. Algae are used both as a food supplement and as an addition to functional food. Meat products, cereal-based products, and fermented functional foods, such as cheeses, are the main products in the market supplemented with algae [7]. Statistics from the 2012 global harvest demonstrated that 38% of the 23.8 million seaweed harvest was used for human consumption, without counting the consumption of hydrocolloids derived from algae as agars, alginates, and carrageenans [1,8]. Currently, more than 10,000 species of algae are known, but only about 200 species are consumed worldwide, with the brown algae species being the most consumed, followed by red algae species and then the green algae species [6,9]. Despite the considerable number of brown algae species consumed worldwide, under the European regulation there are only about 23 brown seaweed species authorized for food applications, which are summarized in Table 1 [10].

Different studies have already demonstrated that brown algae compounds are associated with different pharmaceutical properties that make them useful in the prevention and treatment of a wide spectrum of disorders and/or diseases [11,12,13].

Coronary heart disease (CHD) is the principal cause of mortality in Europe, accounting for 45% of all deaths [14]. The major risk factor for this disease is excessive dietary cholesterol consumption; once arterial plaques have formed, they can result in lethal myocardial infarction, heart attacks, and cerebrovascular diseases [14,15]. High levels of cholesterol are also associated with other diseases, such as cancer, diabetes, and obesity [16]. One of the main approaches to lower blood cholesterol levels is to adhere to a healthy lifestyle by increasing physical activity, avoiding tobacco products, and adopting dietary changes, such as reducing saturated fat intake. These changes in the diet and lifestyle habits are recommendations both before and in conjunction with therapies using cholesterol-lowering drugs [17]. Also, the intake of natural products as supplements is accepted as a strategic therapy in combination with anti-hyperlipidemic drugs [18]. Different sources of compounds have been used for this purpose, namely yeast extracts, herbal extracts, plant sterols, dietary fiber, and also marine algae, specifically different brown algae species [13]. Due to the widespread interest in recent years in algae functional foods [1] and given that brown algae are rich in bioactive compounds and considered a valuable commercial resource [19], the propose of this review is to summarize the present knowledge about the hypocholesterolemic effect of brown algae species and their purified compounds.

## 2. Hypercholesterolemia and the Actual Treatment

Cholesterol is an essential factor for cell homeostasis, having an important role in the synthesis of hormones and bile acids and in membrane structures [20,21]. Whole-body cholesterol homeostasis is a tightly regulated process that involves de novo biosynthesis, dietary cholesterol absorption, and biliary clearance and excretion [16,22]. An imbalance of these processes may lead to hypercholesterolemia, which is characterized by high levels of total cholesterol (Tc), low-density lipoprotein cholesterol (LDL-c), or triglycerides (TGs), and/or a decrease in high-density lipoprotein cholesterol (HDL-c) [23].

**Figure 1:**
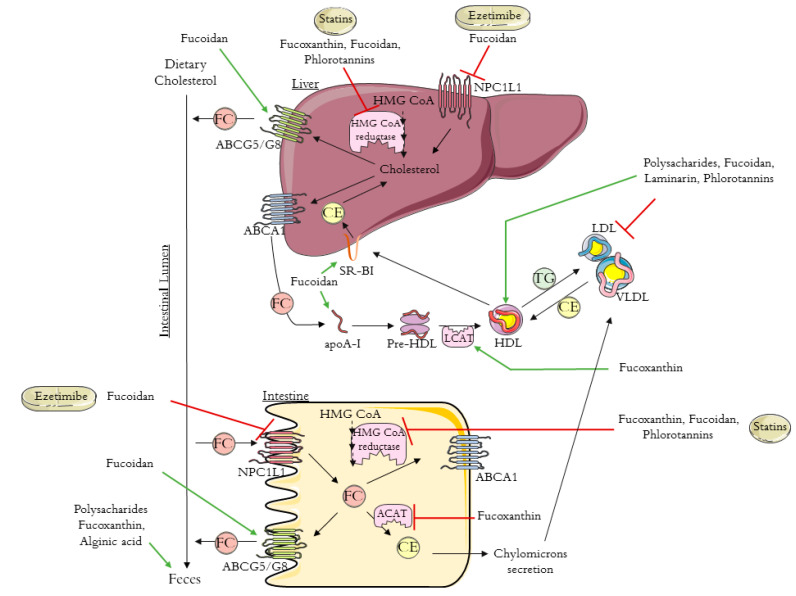
Schematic diagram of reverse cholesterol transport (RCT), intestinal cholesterol absorption, and the mechanisms of action of ezetimibe, statins, and brown algae compounds on cholesterol metabolism (polysaccharides, fucoxanthin, fucoidan, alginic acid, laminarin, and phlorotannins). HMG-CoA: 3-hydroxy-3-methyl-glutaryl coenzyme A; NPC1L1: Niemann–Pick C1-like 1 protein; ABCA1: ATP-binding cassette transporter A1; ABCG5/8: ATP-binding cassette transporters G5/8; SR-BI: scavenger receptor class B type I; ACAT: acyl-CoA:cholesterol acyltransferase; apoA-I: apolipoprotein A-I; HDL: high-density lipoprotein; VLDL: very low density lipoprotein; LDL: low-density lipoprotein; LCAT: lecithin-cholesterol acyl transferase; TG: triglyceride; FC: free cholesterol; CE: esterified cholesterol. The figure was designed using images from Servier Medical Art Commons Attribution 3.0 Unported License. (http://smart.servier.com). Servier Medical Art by Servier is licensed under a Creative Commons Attribution 3.0 Unported License.

Excess cholesterol in tissues is removed by a pathway known as reverse cholesterol transport (RCT) that transports cholesterol from non-hepatic tissues to the liver for metabolism and bile excretion [24]. The principal lipoprotein involved in the cholesterol efflux from peripheral tissues is the HDL-c and its apolipoprotein (apo) A-I, the major protein in HDL particles [25]. Moreover, there are also three important membrane cholesterol transporters that have a major role in the RCT process: ATP-binding cassette (ABC) transporters A1 (ABCA1) and G1 (ABCG1) and scavenger receptor class B type I (SR-B1) [26,27,28]. The ABCA1 transports the cholesterol to apoA-I, leading to the formation of the HDL-c particles (Figure 1). The HDL particles interact with ABCG1 to incorporate more cholesterol, which is then esterified by the enzyme lecithin-cholesterol acyl transferase (LCAT) (Figure 1) [24,25].

Next, the cholesterol is delivered to the liver by a direct or an indirect pathway. In the direct pathway, HDL binds with high affinity to SR-B1 in the liver and transfers its cholesterol content (Figure 1). By the indirect pathway, the esterified cholesterol (CE) is transferred to apolipoproteins B-100 (apoB-100), to very low density lipoprotein (VLDL), or especially to LDL, in exchange for TG molecules (Figure 1) [24,25]. After this process, HDL-c molecules can re-enter the RCT cycle. Based on this knowledge, it has been proven that the plasma concentration levels of the HDL-c are inversely related to the risk of CHD [24,25,29]. The final step in RCT is the most important mechanism for cholesterol body elimination, since the hepatic cholesterol, free or converted into bile acids, is secreted in the bile and then eliminated via the feces (Figure 1) [29,30].

Whole-body cholesterol homeostasis is largely affected by the intestinal cholesterol absorption once its absorption percentage averages 56% [31,32]. Niemann–Pick C1-like 1 protein (NPC1L1) is the key player in dietary cholesterol intake, transporting dietary and bile cholesterol from the intestinal lumen to the enterocyte. Afterwards, the cholesterol can be esterified by acyl-CoA:cholesterol acyltransferase (ACAT), assembled into chylomicrons, and exported into circulation (Figure 1) [33]. Likewise, the unesterified cholesterol can also be exported to the blood stream through the protein transporter ABCA1, localized in the basolateral membrane of the enterocyte (Figure 1) [34]. The unesterified cholesterol is also effluxed back into the intestinal lumen through the transporter proteins localized in the apical membrane of the enterocytes, ABCG5/ABCG8 (Figure 1) [35].

The first approach to preventing cardiovascular disease and lowering cholesterol levels is lifestyle changes together with drug-based therapy [17]. The two main lipid-lowering drugs used are statins and ezetimibe, which act according to different mechanisms, via the de novo cholesterol synthesis and via the dietary cholesterol absorption, respectively [18,20].

Statins are the first-line drugs for lowering cholesterol levels, and there are currently seven different types approved in the United States: simvastatin, atorvastatin, lovastatin, pravastatin, fluvastatin, rosuvastatin, and pitavastatin. Statins bind to the active site of 3-hydroxy-3-methyl-glutaryl coenzyme A (HMG-CoA) reductase, the rate-limiting enzyme in cholesterol biosynthesis, leading to its inhibition (Figure 1) [36,37]. Consequently, statins cause the upregulation of the expression of LDL receptors, improving the clearance of LDL in circulation [23,33]. It was previously demonstrated that statins increase HDL levels by 5% to 10% [38]. The mechanism that leads to this increase is still not very clear, however, it is already known that some statins improve ABCA1 expression and hepatic apoA-I production, which consequently increase HDL-c formation, leading to the association of statin therapy with enhanced RCT [38,39,40]. Despite the good results obtained with statins therapy, these drugs can cause some adverse effects. There are cases for which the statins treatment is not recommend; for example, some people are intolerant to statins, specifically patients with high cardiovascular risk. One approach to avoid the side effects of statins is to use the combination of statins with non-statin lipid-modifying drugs.

Ezetimibe (trademark name Zetia), the first drug approved after statins, is a potent intestinal cholesterol absorption inhibitor that targets NPC1L1 [20,41]. This therapy also presents good results in terms of decreasing LDL-c [42]. Beyond the use of ezetimibe alone, another treatment approach to primary hyperlipidemia is its use in combination with statins, which has a synergistic effect with greater results for the reduction of blood Tc and LDL-c [42].

To prevent hypercholesterolemia and complement the drug treatments with statins, ezetimibe, or other drugs, it is important to look for new potent drugs and/or new therapeutic strategies, such as the use of dietary supplements derived from natural products [16].

## 3. The Anti-Hypercholesterolemic Effect of Brown Algae

The search for new drugs, nutraceuticals, and dietary supplements based on natural compounds has led to a growing interest in the study of the composition and bioactivity of marine algae [2,43]. Around the world, there are about 1800 species of brown algae (Phaeophyta), showing a variety of size and forms range from small filamentous to large and complex structures [44,45]. Most species of brown algae are exclusively found in oceans dispersed in cold waters along continental coasts and can also be found attached to rocky coasts in temperate zones or floating freely [46,47]. Just 1% of brown algae species can be found in freshwater, which are usually attached to rocks [47]. Brown algae species contain a variety of compounds, such as proteins, minerals, vitamins, alkaloids, sterols, fatty acids, carotenoids, non-digestible polysaccharides, and phenolic compounds, mostly phlorotannins [48,49]. However, the composition of algae is greatly affected by the geographic and atmospheric conditions of their habitat [50]. It is also necessary to take into account that post-harvesting storage and processing, such as extraction using different solvents (water, organic solvent, water–organic solvent mixtures) can also affect the composition of the obtained algae extract [51]. These algae species have been intensively studied not only for their anti-hyperlipidaemic activity [52,53], but also for their anti-tumorigenic [54,55], anti-diabetic [56,57], antiviral [58], anti-inflammatory [59,60], and antioxidant [48,60] capacities, among others.

Different in vivo studies, summarized in Table 2, have reported the hypocholesterolemic effect of different brown algae species [10,14,52,53,54,55,56]. In studies where brown algae extracts were used for diet supplementation or in oral administration, it was reported that, in general, there is a decrease in Tc, TG, and LDL-c levels and an increase in HDL-c levels. In these reports, the effects of brown algae on lipid levels were associated with a possible impact on the cholesterol biosynthesis due to a modulation effect on the high affinity receptor of lipoprotein metabolism [53,61]. In addition, brown algae have been reported to have the capacity to increase cholesterol excretion in feces due to the ability of the algae compounds to bind dietary cholesterol [62]. The mechanisms for the cholesterol-lowering effects of brown algae have not been clarified yet, but have often been associated with the presence of the diversity of compounds identified in these species, which led to the study of the hypocholesterolemic effect of isolated compounds obtained from different brown algae species. The most studied brown algae compounds in this area have been carotenoids, polysaccharides, and phlorotannins, and more recently interest has emerged in the hypocholesterolemic potential of alga-derived proteins and peptides.

### 3.1. Carotenoids—Fucoxanthin

**Figure 2:**
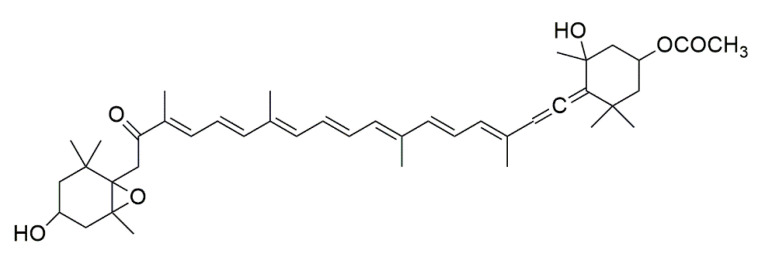
Fucoxanthin structure [67].

Carotenoids are one of the major compounds that contribute to the antioxidant capacity of seaweeds [55]. The most abundant carotenoid in brown algae is fucoxanthin. Different studies have reported that fucoxanthin has a unique structure (Figure 2), with an allenic bond, a 5,6-monopoxide, and nine conjugated double limits, which is a determinant for the different biological activities that characterize this compound [55,65,66]. Fucoxanthin presents different biological activities, such as antioxidant, anti-inflammatory, antidiabetic, antitumor, anti-obesity, hypolipidemic, and hepatoprotective activity [57,66,67].

As for the hypolipidemic effect of fucoxanthin, different studies have reported that this compound has the capacity to decrease the levels of hepatic cholesterol and TGs, affecting both the cholesterol synthesis and absorption [68,69]. These studies reported that fucoxanthin led to the inhibition of HMG-CoA reductase, inhibiting cholesterol synthesis [68,69]. Likewise, fucoxanthin was seen to affect cholesterol absorption by increasing the fecal cholesterol and TGs and by decreasing the Acyl-CoA:cholesterol acyltransferase (ACAT) mRNA level while increasing LCAT mRNA levels; both these enzymes catalyze the esterification of free cholesterol into cholesterols ester (Figure 1) [68].

### 3.2. Polysaccharides

Seaweeds are known to have a significant amount of polysaccharides that are structural components of the cell wall [70]. This group of compounds has been intensively studied due to their commercial usage in different areas, namely food, pharmaceutical, and cosmetic industries [2]. Different characteristics of polysaccharides, such as their molecular size, the type of glycosidic bond, and the type and ratio of constituent monosaccharides, are related to the different biological activities of these compounds [70]. In general, it has been reported that polysaccharides present anti-inflammatory [2], anti-viral [2,70], antioxidant [70], antitumor [2], anticoagulant [2,71], and hypolipidemic activities [2,70], among others. Brown algae contain different polysaccharides, such as alginates, fucoidans, cellulose, and laminarins [48,72].

The hypocholesterolemic effect of polysaccharides extracts and specific sulphated polysaccharide extracts isolated from different brown algae have been studied and are reviewed in Table 3. Algae polysaccharides have shown the capacity to decrease blood lipid and Tc levels, while increasing HDL-c levels (Figure 1) [65,73,74,75]. It has been reported that polysaccharides from brown algae decrease cholesterol absorption, as these compounds have the capacity to bind dietary cholesterol, enhancing fecal cholesterol excretion (Figure 1) [63]. Cholesterol absorption is also greatly affected by the alginic acid that exists in large amounts in brown algae. Sodium alginate in algae is converted into free alginic acid, forming a gel that is not absorbed in the small intestine and binds to dietary cholesterol, increasing its excretion (Figure 1) and, consequently, reducing the amount of cholesterol absorbed in the intestinal lining [76].

**Figure 3:**
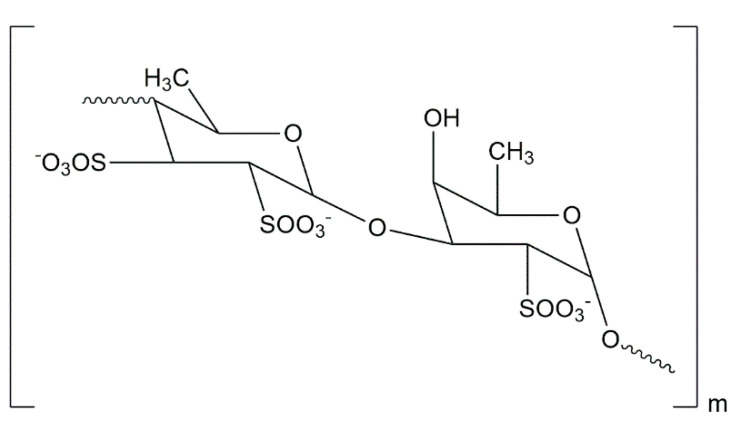
Fucoidan structure [77].

The predominant fucan present in brown algae is fucoidan [70]. The major building block of fucoidan is L-fucose-4-sulfate, which is presented in Figure 3, but the fucoidan structure varies significantly with the seaweed species and the extraction method [71].

Fucoidan has been extensively studied, and different biological activities have previously been reported for this compound, including anti-inflammatory, antiviral, antitumor, immunomodulatory, and also hypolipidemic activity [70,78,79,80]. As summarized in Table 3, the anti-hypercholesterolemic effect of the fucoidan isolated from different algae has shown the capacity to lower serum lipid levels [59,79,80,81,82]. Furthermore, fucoidan has been associated with an improvement in the initial step of RCT, since this compound leads to higher levels of apoA-I and lower levels of apoB, which consequently improves cholesterol clearance (Figure 1) [27]. The same study reported that fucoidan accelerate lipid transfer from plasma to the liver through the upregulation of SR-B1 and HDL (Figure 1). Fucoidan has also been shown to lead to a decrease in cholesterol absorption by decreasing NPC1L1 expression, like ezetimibe, and increasing cholesterol excretion by upregulating ABCG8 transporter (Figure 1) [27]. Besides all of these characteristics, fucoidan also affects cholesterol synthesis by reducing the mRNA expression of HMG-CoA reductase enzyme, an effect similar to statins (Figure 1) [81].

**Figure 4:**
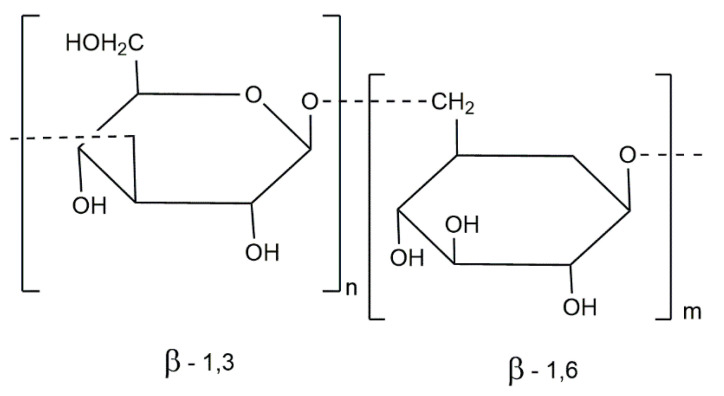
Laminarin structure [77].

The most abundant storage polysaccharide present in brown algae is laminarin. This water-soluble polysaccharide consists of β-(1-3)-glucan with β-(1-6)-glycosidic bonds of 20–25 units (Figure 4) and can contain mannitol or glucose residues as reducing ends [83].

Laminarin has different biological activities, including anti-inflammatory, anticoagulant, antioxidant, anticancer, and hypolipidemic activity [71,76,81]. As summarized in Table 3, laminarin decreases TG, Tc, and LDL-c levels and increases HDL-c levels (Figure 1) [83,84]. Nevertheless, the hypocholesterolemic effect and the mechanism of action of laminarin isolated from brown algae need to be further investigated.

### 3.3. Phlorotannins

The principal class of phenolic compounds that constitute up to 25% of the dry weight of brown algae is the phlorotannins [85]. This class of tannins is constituted by the polymerization of a variable number of phloroglucinol units, presenting a molecular mass between 126 and 650,000 Da [85,86]. Phlorotannins are highly hydrophilic components of algae cell walls, and, as a secondary function, are defense metabolites that respond to abiotic and biotic stress conditions [72,86].

In recent years, different phenolic compounds, including phlorotannins, have been intensively studied, and it has been clearly demonstrated that this group of compounds has different biological activities, such as antioxidant, antibacterial, anti-inflammatory, anti-diabetes, anticancer, anti-HIV, and hypolipidemic activity [87,88].

The hypolipidemic activity of phlorotannins and their mode of action have not yet been completely described, but it has already been demonstrated that these compounds affect the biosynthesis and absorption of cholesterol [13]. Polyphenol-rich extract of *Fucus vesiculosus* and *Ecklonia cava* have showed the capacity to inhibit the cholesterol absorption (Table 4) [89,90], however, its mode of action is still not clear. In fact, previous studies suggested that the *Ecklonia cava* may have the capacity to inhibit the cholesterol esterase, since the inhibition of this enzyme limits the absorption of dietary cholesterol [10]. NPC1L1 transporter is a key player in cholesterol absorption, and it is already know that phenolic compounds isolated from plants/fruits have the capacity to inhibit NPC1L1 expression, presenting a mode of action similar to ezetimibe [91,92,93].

**Figure 5:**
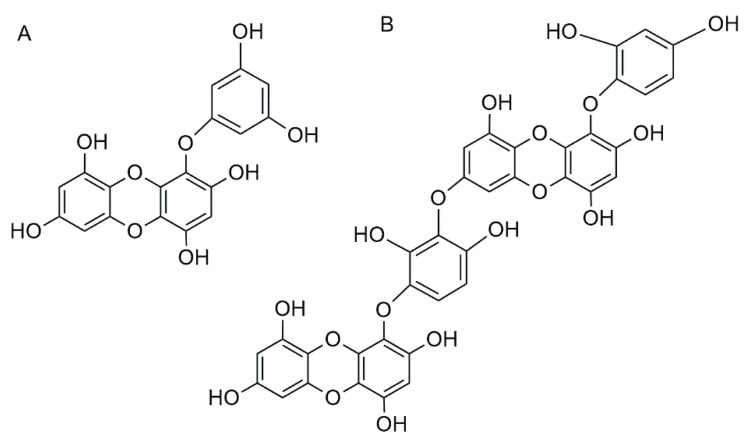
Chemical structure of (**A**) eckol and (**B**) dieckol.

As summarized in Table 4, the rich phlorotannin extracts and the purified phlorotannins eckol and dieckol lead in most cases to the regulation of serum lipid levels, decreasing LDL-c and increasing HDL-c expression. It has been suggested that there is a possible relationship between the degree of polymerization of phlorotannins and the reduction in serum lipid levels, since dieckol, a hexamer, presented a stronger hypolipidemic effect than eckol, which is a trimer (Figure 5) [13]. Other studies have reported that the hypolipidemic effect of phlorotannins may also be related to the antioxidant, anti-inflammatory, and hepatoprotective activities of these compounds, leading to the suppression of fat-induced hepatic damage and, consequently, blood lipid normalization [13,52]. Furthermore, it has been reported that phlorotannin-rich extracts of *F. vesiculosus* and *Ecklonia cava* as well as purified dieckol have the capacity to inhibit the HMG-CoA reductase enzyme, affecting cholesterol synthesis [90,94], thus demonstrating that phlorotannins can act with the same mode of action as statins.

### 3.4. Proteins/Peptides

Proteins compose 5% to 15% of the dry weight of brown algae [95]. Comparing the composition, structure, and bioactivities of algae protein with other algal compounds, little is known about the proteins [96]. The proteins isolated from different seaweeds belong to the two main groups of functionally active proteins: phycobiliproteins and lectins [96]. It has been reported that protein bioactive peptides with different activities were obtained by hydrolysis of macroalgae [95]. The hydrolysates of proteins from different brown algae generate peptides showing antioxidant, antibacterial, antihypertensive, and angiotensin-converting enzyme (ACE) inhibitor capacity [95,96]. However, little is known about the hypocholesterolemic effect of peptides derived from brown algae. A previous study reported that *F. vesiculosus* extract rich in phlorotannins and peptides led to cholesterol absorption inhibition as well as to HMG-CoA reductase inhibition (Table 4) [90]. Another study, but with the red algae (*Porphyra yezoensis*), reported that peptides isolated from this seaweed present the capacity to reduce plasma and hepatic cholesterol [95,96]. However, further studies are needed to understand the effect of proteins and peptides isolated from brown algae on the regulation of cholesterol levels.

## 4. Conclusions

With this review on the most recent research on the anti-hypercholesterolemic effect of brown algae and their isolated compounds, it is possible to conclude that brown algae are a valuable food source of natural compounds that can be used for hypercholesterolemic therapy. Different brown algae species have showed the capacity to lower blood lipid levels and total cholesterol, as demonstrated in numerous different studies [52,53,54,55,56]. In the case of the polysaccharides isolated from brown algae, it has been demonstrated that these compounds essentially affect cholesterol absorption by increasing cholesterol excretion [23,55,68]. As for phlorotannins, different reports demonstrated that its effect in lowering cholesterol is not only because it affects the absorption and excretion of cholesterol, but also because it affects its biosynthesis [84,88]. Brown algae are also rich in proteins and bioactive peptides, but little is known about the effect and the mode of action of these compounds in hypercholesterolemic situations.

Although there have already been several studies on the hypocholesterolemic effect of brown algae extracts and different studies with isolated compounds, further studies are needed in order to understand in detail the mode of action in the different proteins/transporters/lipids involved in the cholesterol metabolism. It is also important to study if the compounds from the algae that are reported to have the capacity to affect the expression of different proteins associated with cholesterol metabolism can permeate the intestinal barrier and reach their target.

Given the already studied effect of brown algae on cholesterol reduction, and also given that the different therapies to lower blood cholesterol levels, either with statins or with ezetimibe, have associated side effects, the use of brown algae as a complementary treatment should be considered. For this, it will be necessary to study in more detail the consumption effects associated with brown algae and to study possible interactions and side effects of brown algae and their purified compounds when consumed simultaneously with prescribed drugs.

## Figures and Tables

**Table 1:** List of brown algae species for human food applications in Europe under the regulation (UE) 2015/2283 [10].

Brown Algae Species
*Ascophyllum nodosum*
*Alaria esculenta*
*Eisenia bicyclis*
*Fucus vesiculosus*
*Fucus serratus*
*Fucus spiralis*
*Himanthalia elongata*
*Laminaria digitata*
*Saccharina japonica*
*Saccharina latissima*
*Saccharina longicruris*
*Sargassum fusiforme*
*Undaria pinnatifida*

**Table 2:** Overview of the in vivo hypocholesterolemic effect of different brown algae species under different extraction conditions.

Brown Algae Specie	Algae Preparation and Administration Mode	Effect	Ref.
*Ecklonia stolonifera*	Oral administration of EtOAc and n-BuOH fractions derived from EtOH seaweed extract at a dose of 100 mg/kg of body weight	↓ Tc, TGs, LDL-c ↑ HDL-c	[13]
*Ecklonia cava*	Capsules, twice per day (200 mg seaweed powder per tablet)	↓ Tc, LDL-c	[18]
*Iyengaria stellata*	Oral administration of ethanol extracts suspended in distilled water at 10 mg/200 g of body weight	↓ Tc, TGs, LDL-c ↑ HDL-c	[53]
*Colpomenia sinuosa*	Oral administration of ethanol extracts suspended in water at 10 mg/200 g of body weight	↓ Tc, TGs, LDL-c ↑ HDL-c	[53]
*Spatoglossum asperum*	Oral administration of ethanol extracts suspended in distilled water at 10 mg/200 g of body weight	↓ Tc, TGs, LDL-c ↑ HDL-c	[53]
*Spatoglossum asperum*	Diet supplemented with oily fractions of seaweed at 10 mg/200 g of body weight	↓ Tc, TGs, LDL-c	[61]
*Heterochordaria abietina*	Diet supplemented with 5% seaweed powder	↓ Tc, LDL-c	[62]
*Sargassum micracanthum*	Diet supplemented with 5% seaweed powder	↓ Tc, TGs	[62]
*Sargassum patens*	Diet supplemented with 5% seaweed powder	↓ Tc, LDL-c	[62]
*Cystoseira sisymbrioides*	Diet supplemented with 5% seaweed powder	↓ Tc, free cholesterol, LDL-c	[62]
*Laminaria diabolica*	Diet supplemented with 5% seaweed powder	↑ HDL-c	[62]
*Sargassum ringgoldianum*	Diet supplemented with 5% seaweed powder	↑ HDL-c	[62]
*Padina arborescens*	Diet supplemented with 5% seaweed powder	↑ HDL-c	[62]
*Sargassum polycystum*	Diet supplemented with 5% seaweed powder	↓ Tc, TGs, LDL-c ↑ HDL-c	[63]
*Undaria pinnatifida*	Diet supplemented with 100 g of seaweed powder/kg of body weight	↓ Tc, TGs, LDL-c ↑ HDL-c	[64]

Tc: total cholesterol; TGs: triglycerides; LDL-c: low-density lipoprotein cholesterol; HDL-c: high-density lipoprotein cholesterol.

**Table 3:** Overview of the in vitro and/or in vivo hypocholesterolemic effect of polysaccharides isolated from different brown algae species under different extractions conditions.

Isolated Compound	Model Used	Effect	Ref.
Sulfated polysaccharides (*Cystoseira crinite*)	in vivo	↓ Tc, TGs, LDL-c ↑ HDL-c	[73]
Sulfated polysaccharides (*Sargassum polycystum*)	in vivo	↓ Tc, TGs, LDL-c ↑ HDL-c	[74]
Polysaccharide extract (*Fucus vesiculosus*)	in vivo	↓ Tc, TGs ↑ HDL-c	[75]
Polysaccharide extract (*Laminaria japonica*)	in vivo	↓ Tc, TGs, LDL-c, HDL-c	[65]
Sodium alginate	in vivo	↑ Cholesterol excretion	[76]
Fucoidan *(Ascophyllum nodosum*)	in vivo	↓ LDL-c, apoB, NPC1L1 ↑ SR-B1, apoA-I, ABCA1, ABCG8	[27]
Fucoidan *(Fucus vesiculosus)*	in vivo	↓ Tc, TGs, LDL-c ↑ HDL-c	[81]
	in vitro	↓ HMG-CoA mRNA, LDL receptor	
Fucoidan (*Saccharina Japonica)*	in vivo	↓ TGs, oxidative-LDL	[59]
Fucoidan (*Laminaria Japonica)*	in vivo	↓ Tc, TGs, LDL-c ↑ HDL-c, LCAT	[80]
Fucoidan (*Sargassum Henslowianum)*	in vivo	↓ Tc, TGs, LDL-c	[82]
Fucoidan (*Cladosiphon okamuranus)*	in vivo	↓ Tc, TGs, LDL-c ↑ HDL-c	[79]
Laminarin (*Eisenia bicyclis*)	in vivo	↓ Tc, TGs, LDL-c ↑ HDL-c	[84]

TC: total cholesterol; TGs: triglycerides; LDL-c: low-density lipoprotein cholesterol; HDL-c: high-density lipoprotein cholesterol.

**Table 4:** Overview of the in vitro and/or in vivo hypocholesterolemic effect of phlorotannins isolated from different brown algae species under different extractions conditions.

Isolated Compound	Model Used	Effect	Ref.
Phlorotannin ethyl acetate fraction (*Ecklonia stolonifera*)	in vivo	↓ Tc, TGs, LDL-c ↑ HDL-c	[13]
Phlorotannin n-butanol fraction (*Ecklonia stolonifera*)	in vivo	↓ Tc, TGs, LDL-c ↑ HDL-c	[13]
Polyphenol extract (*Ecklonia cava*)	in vivo	↓ Tc, LDL-c ↑ HDL-c	[89]
Phlorotannin and peptide-rich extract (*Fucus vesiculosus*)	in vitro	↓ HMG-CoA reductase activity ↓ Cholesterol absorption	[90]
Seapolynol™ (*Ecklonia cava*)	in vivo	↓ Tc, TGs, LDL-c	[52,94]
	in vitro	↓ HMG-CoA reductase activity	[94]
Eckol	in vivo	↓ Tc, TGs, LDL-c	[13]
Dieckol	in vivo	↓ Tc, TGs, LDL-c ↑ HDL	[13,94]
	in vitro	↓ HMG-CoA reductase activity	[94]

TC total cholesterol; TGs: triglycerides; LDL-c: low-density lipoprotein cholesterol; HDL-c: high-density lipoprotein cholesterol.
